# Epigenetic aging in oocytes

**DOI:** 10.18632/aging.204976

**Published:** 2023-08-05

**Authors:** Peera Wasserzug-Pash, Michael Klutstein

**Affiliations:** 1 Institute of Biomedical and Oral Research, Faculty of Dental Medicine, The Hebrew University of Jerusalem, Jerusalem 9112001, Israel

**Keywords:** oocytes, heterochromatin, epigenetics, aging, maturation

Aging-related phenotypes span many different tissues and cell types, and start to occur at different ages- a different typical age for every cell type. Among the earliest occurring aging events in the human body is the beginning of female reproductive aging and deterioration. The clinical cut-off for advanced maternal age (AMA), a condition associated with poor reproductive outcomes, is 35 years old. The early onset of reproductive aging poses a significant challenge to clinicians since a global consistent increase in maternal age at first birth has occurred in recent decades, effectively shortening the available time window for reproduction [1]. As the rate of patients with advanced maternal age rises, and with it, the number of patients in fertility clinics, so does the necessity for a fundamental understanding of the reproductive aging process. In recent years, it has been established that there is a substantial dominating influence of oocyte quality loss on age-related fertility decline. This is best demonstrated by the rise in IVF success rates in reproductively aged women when they receive an egg donation from a younger woman [2]. Oocyte quality loss is characterized by diminished cellular function and an increased occurrence of chromosomal non-disjunctions. The emergence of aneuploidy is a well-known feature of oocyte aging, a subject that has yielded several intriguing insights into the aging process of cell cycle regulation and chromosomal segregation machinery [3]. However, oocyte quality decline occurs even before the onset of aneuploidy. This decline is characterized by a decrease in oocyte quantity, poor maturation rates, and a decline in pregnancy outcomes. As for today, the bio-molecular environment of the early-aging oocyte and how it manifests in oocyte loss of function are poorly understood.

Our recent publication [4] addresses the question of additional, epigenetic mechanisms that lead to the occurrence of age-related oocyte quality loss. Epigenetic aging is a broadly studied well-characterized phenomenon. It has been shown in many cell types and biological systems, that with age, there is a decline in heterochromatin levels, a shift in DNA methylation, and deterioration in chromatin architecture. Heterochromatin loss and epigenetic aging play an important role in the aging process, interacting with other features of cell and tissue aging [5]. In Wasserzug-Pash et al., we show that the loss of heterochromatin also occurs in mouse oocytes, at an age before the onset of significant aneuploidy (9 months). This loss of heterochromatin with age also occurs in human meiotically arrested oocytes, donated by IVF patients. The nature of heterochromatin loss includes several types of heterochromatin (constitutive-H3K9me2 and HP1, and facultative- H3K27me3), and extends throughout several oocyte growth stages (primordial follicles, antral follicles, fully grown follicles, meiotic oocytes during ovulation). Importantly, we show that the loss is specific to heterochromatin, and does not apply to euchromatic markers such as H3K27Ac and H3K4me3. This negates an interpretation where whole nucleosomes are lost from older oocytes' chromatin.

In a second stage of this project- we show that aged mouse oocytes lose their control over RNA transcription- showing elevated levels of dsRNA and Dicer- both markers of transcriptional control. We also investigated the levels of retrotransposon transcripts- which are elevated in aged oocytes, as well as Line1 retrotransposon protein L1ORF1p. Consequently, older oocytes show elevated levels of DNA damage markers such as Rad51 and gamma-H2AX.

To causally link the two phenomena, we have disrupted heterochromatin structure in young (2 months old) oocytes using two known heterochromatin damaging agents- Chaetocin and TSA. As a result, the treated oocytes showed elevated retrotransposon transcription, elevated retrotransposon protein synthesis, elevated DNA damage, and reduced maturation ability *in-vitro* compared to untreated oocytes.

Finally, to demonstrate the central role of epigenetic changes in oocyte aging we show that the reversal of heterochromatin loss leads to a dramatic improvement in oocyte quality and function. Using heterochromatin enhancing small molecules or overexpression of heterochromatin genes (Ezh2, Sirt1), we elevated heterochromatin levels in old (9 months) mouse oocytes. This led to a decrease in retrotransposon expression levels and a decrease in DNA damage. It also led to a dramatic increase (2 fold) of *in-vitro* maturation ability in these old oocytes. Interestingly, the same result is achieved when retrotransposon activity is blocked by inhibiting reverse-transcriptase activity using AZT. This result shows that retrotransposon activation is a significant event during oocyte epigenetic aging.

Our work reveals unknown aspects in oocyte aging. It mainly offers two substantial and innovative advances: first, it investigates chromatin aging processes in the early stages of oocyte aging. The distinction of early aging allowed us to define and analyze previously unknown molecular characteristics of early-aging oocytes. In oocyte aging research, we can use these insights to better understand the damage and deterioration linked to later stages of aging. By following the escalation of the mild events described in this paper, we could determine how they lead to the total loss of function that defines the later stages of oocyte aging. The aspect of heterochromatin loss is a great example in this context- heterochromatin is a dynamic and important component of chromatin, that influences many aspects of the cellular environment through direct or indirect interactions, including cytoskeleton, spindle establishment, oocyte-to-zygote transition, RNA processing and transcription, and more. Heterochromatin loss during the early aging period could explain the deterioration of these systems later in the aging timeline. The second important insight provided in this paper is the concrete aging pathway it describes. Starting with a molecular event of heterochromatin loss, we identify downstream events that lead to the outcome of maturation failure. Tracking the direct chain of events allowed us to not only better understand the molecular features of oocyte aging, but also to therapeutically target them. If this approach can be applied in the IVF clinic, it will provide a concrete therapy option for age-related fertility loss, which is currently unaddressed in the clinic.

In summary, our group demonstrates basic principles in the early aging of mammalian oocytes. This demonstration provides us with an improved understanding of how oocyte aging processes are initiated, and which events lead to deleterious outcomes, damaging oocyte maturation and quality.
